# Cardiopulmonary nerve stimulation as a novel therapy for cardiac autonomic nervous system modulation

**DOI:** 10.3389/fnins.2024.1377171

**Published:** 2024-02-28

**Authors:** Siamak Salavatian, Julio C. Spinelli, Jeremy A. Schaefer, Imad Libbus, Aman Mahajan, J. Andrew Armour

**Affiliations:** ^1^Department of Anesthesiology and Perioperative Medicine, University of Pittsburgh, Pittsburgh, PA, United States; ^2^Department of Medicine, Division of Cardiology, University of Pittsburgh Medical Center, Pittsburgh, PA, United States; ^3^Cardionomic, Incorporated, New Brighton, MN, United States

**Keywords:** cardiopulmonary nerve stimulation, neuromodulation therapy, heart failure, cardiac autonomic nervous system, autonomic imbalance

## Introduction

Acute decompensated heart failure (ADHF) carries a significant burden of mortality and morbidity, with few effective treatments available (Heidenreich et al., 2022; Greene et al., 2023). Cardiopulmonary nerve stimulation (CPNS), which involves targeted electrical stimulation of specific nerves that innervate the heart, is an innovative emerging therapeutic ADHF management strategy (Petru et al., 2020; Goedeke et al., 2022; Emani et al., 2023). In a recent study, Emani et al. (2023) used the CPNS using a low-level stimulation which enhanced cardiac inotropy, decreased energy consumption, and improved patients' symptoms, function, and quality of life (Emani et al., 2023). The mechanism of action of CPNS that drove these promising results was not described by Emani et al. (2023).

This commentary aims to explore the mechanisms of CPNS, focusing on its neuromodulatory effects on the cardiac autonomic nervous system (CANS) and its potential to revolutionize ADHF therapy.

## Cardiac autonomic nervous system

**Figure 1:**
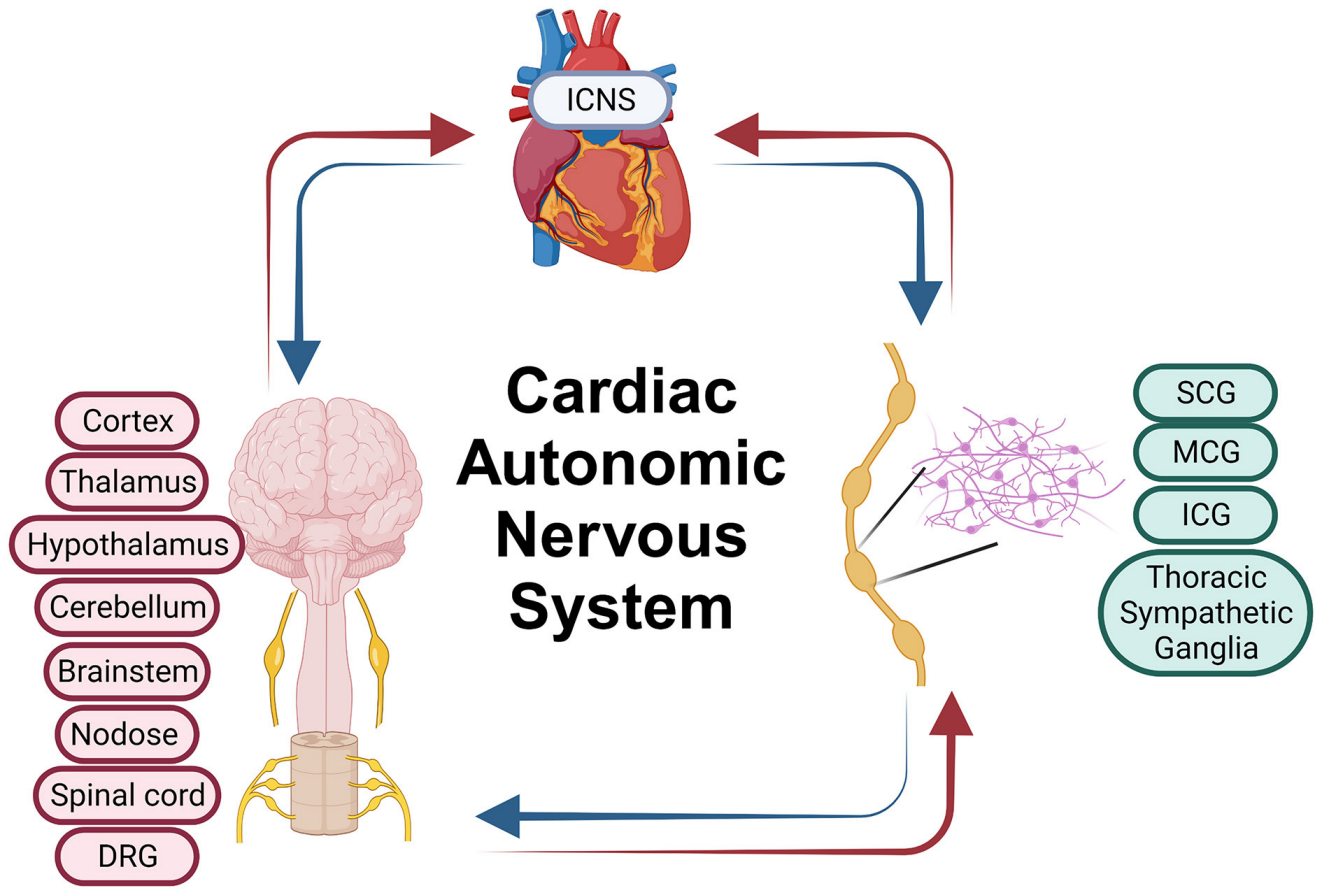
Cardiac autonomic nervous system. ICNS, Intrinsic Cardiac Nervous System; SCG, Superior Cervical Ganglia; MCG, Middle Cervical Ganglia; ICG, Inferior Cervical Ganglia; DRG, Dorsal Root Ganglia. Parts of this figure were created with BioRender.com.

The CANS is a complex network of multiple layers of neural control, each hierarchically arranged to regulate cardiac function (Armour and Ardell, 2004; Hadaya and Ardell, 2020). CANS functionality is underpinned by several key layers: (1) the intrinsic cardiac nervous system (ICNS) for local control, (2) intrathoracic extracardiac ganglia, including sympathetic and superior cervical ganglia, for intrathoracic regulation, (3) the spinal cord for processing cardiac sensory information and directing sympathetic outflow, (4) the brainstem for integrating sensory information and modulating autonomic output, and (5) the higher brain centers like the cortex for overarching control (Figure 1).

Neuromodulation of the CANS can be achieved by altering the activity of cardiac neurons or nerves at various levels within the system, each of which can directly or indirectly influence cardiac function.

## CANS stimulation

### Sympathetic nerve stimulation

Activating the sympathetic nervous system releases norepinephrine (NE) in the heart, enhancing contractility, blood pressure, and heart rate. The effect varies depending on the heart region innervated. For example, stimulating the stellate ganglion, which is the fusion of the inferior cervical ganglia and thoracic 1(T1) sympathetic ganglia, on the left side primarily increases left ventricular contractility without significantly affecting heart rate, whereas right stellate ganglion stimulation predominantly elevates heart rate (Ajijola et al., 2015; Dacey et al., 2022). The ability of sympathetic nerve stimulation in altering hemodynamic parameters such as heart rate and contractility is influenced by the stimulation settings, the state of the heart, and the overall condition of the CANS. In heart failure, CANS dysfunction may alter expected responses, with the degree of response variability tied to the extent of CANS remodeling (Tjeerdsma et al., 2001; Campelo and Abreu-Lima, 2004; Hoyer et al., 2008; Kardesoglu et al., 2011; Kishi, 2012; Phillips, 2012; Ali et al., 2018; Hadaya and Ardell, 2020).

### Parasympathetic nerve stimulation

Activating parasympathetic nerve fibers typically leads to bradycardia and may also lower blood pressure. The choice of stimulation parameters and the specific stimulation location are crucial, as they greatly influence the outcomes. For example, stimulating the vagus nerve, a key element of the parasympathetic nervous system, at both the cervical and heart levels leads to bradycardia. However, cervical vagus nerve stimulation is linked to various off-target effects that are absent when only the cardiac branch of the vagus nerve is stimulated (Fitchett et al., 2021). As with sympathetic stimulation, the extent of bradycardia and hemodynamic shifts induced by parasympathetic stimulation is contingent upon the heart's condition and the functional state of the CANS. This variability underscores the complex interplay between heart health and autonomic regulation.

### Afferent nerve stimulation

#### Direct

Stimulating afferent fibers directly can yield various outcomes, dependent on the specific fiber type and stimulation intensity. These effects range from potentially cardioprotective impacts (Fallen, 2005), to adverse reactions like pain, arrhythmias, bradycardia, tachycardia, and other hemodynamic shifts. The diversity of these effects illustrates the complexity and sensitivity of afferent nerve responses to direct stimulation (Schwartz and Foreman, 1991; Chandler et al., 1993).

#### Indirect

The nuanced effects of indirectly modulating afferent neurons are often overlooked in neuromodulation therapies. It's crucial to recognize that the CANS operates as a closed-loop system. As such, sympathetic or parasympathetic stimulation affects more than just contractility, blood pressure, or heart rate; it also alters cardiac sensory neurons. These neurons are responsible for detecting mechanical and chemical changes in cardiac tissues and relay this sensory information throughout the CANS hierarchy for further processing (Armour, 2004; Armour and Ardell, 2004). Upon processing this sensory data, the CANS can undergo a complete state alteration. This underscores the profound impact of sensory input on the autonomic system's overall functioning, highlighting the intricate feedback mechanisms within the CANS (Armour, 2004; Armour and Ardell, 2004).

### Stimulation parameters

In electrical stimulation, parameters such as frequency, amplitude, pulse width, duty cycle, polarity, burst time, and ramp time are critical in shaping therapeutic outcomes. Different stimulation parameters can result in markedly different effects. For example, low-intensity parasympathetic stimulation may offer cardioprotective benefits, whereas high-intensity stimulation of the parasympathetic nerve can precipitate arrhythmias (Ardell et al., 2017; Wang et al., 2019; Kharbanda et al., 2022). Identifying optimal stimulation parameters for maximal therapeutic effects in various cardiac diseases presents a significant challenge. There is a need for extensive translational research involving large animal models to determine these optimal parameters and to thoroughly understand the mechanisms behind a neuromodulation therapy's effectiveness.

### Ganglionated plexi stimulation

The stimulation of cardiac ganglionated plexi (GPs) can lead to diverse outcomes, largely dependent on the types of neurons and nerve fibers present within these GPs. Activation of GPs containing sympathetic, parasympathetic, or local circuit neurons can result in various cardiac responses, including bradycardia, tachycardia, a combination of both, atrioventricular (AV) block, and atrial arrhythmias. This underscores the critical need for precision in targeting specific neuronal GPs in cardiac neuromodulation (Butler et al., 1990; Cardinal et al., 2009; Lim et al., 2011a,b). Stimulating multiple neuron types simultaneously within the GPs may lead to inconsistent and undesirable effects across different subjects. This variability highlights the complexity and challenges in achieving uniform therapeutic responses through GP neuromodulation (Butler et al., 1990; Cardinal et al., 2009).

## CANS imbalance in HF patients

In early HF, the sympathetic nervous system becomes overly active as a compensatory response to maintain cardiac output, driven by heightened sensory signaling from cardiac afferent neurons. As HF progresses without resolution, these neurons continuously signal the CANS for increased sympathetic and reduced parasympathetic activity, leading to autonomic imbalance. This ongoing sympathetic dominance and parasympathetic dysfunction contribute to the worsening of HF, increased arrhythmias, decompensation, and mortality risk (Grassi et al., 1995; Schnabel and Bohm, 1999; Noble, 2000; Floras, 2003, 2009; Borovac et al., 2020; Gronda et al., 2022).

## Cardiopulmonary nerve stimulation

### Location

**Figure 2:**
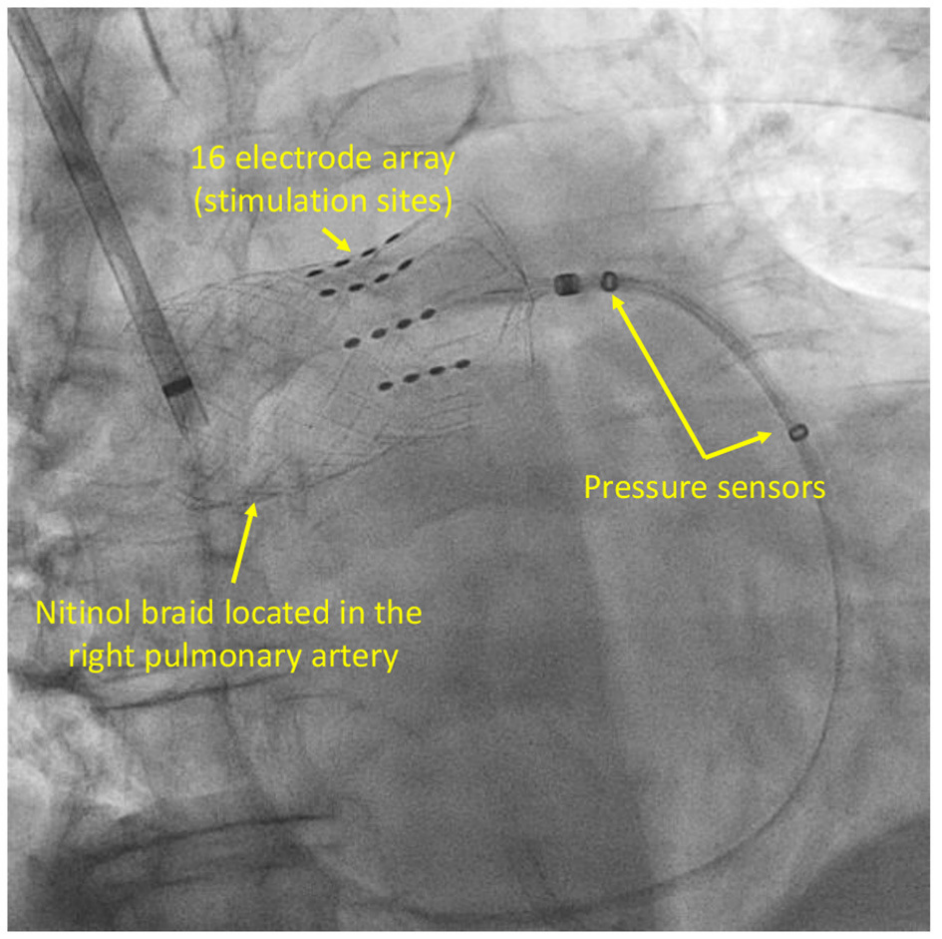
Cardiopulmonary nerve stimulation catheter.

In human clinical studies, CPNS was delivered through a catheter equipped with a 16-electrode anchoring nitinol braid and placed within the right pulmonary artery (RPA; Figure 2). This specific location facilitated targeted stimulation of cardiac nerves in the mid- RPA region (Petru et al., 2020; Goedeke et al., 2022). The RPA, targeted in CPNS, is characterized by a complex mix of nerve structures, including various sympathetic, parasympathetic, and afferent nerves, alongside GPs. This area encompasses a variety of nerves such as the right dorsal medial cardiopulmonary nerve (CPN), right dorsal lateral CPN, right stellate CPN, CPN plexus, both superficial and deep cardiac plexus, vagal cardiac branches, inferior cervical cardiac nerves, and RPA GP (Janes et al., 1986; Murphy and Armour, 1992; Chiou et al., 1997; Kawashima, 2005; Niu et al., 2007; Wang et al., 2010; Wink et al., 2020; Zandstra et al., 2021).

### CPNS effect

CPNS therapy, applied in areas densely populated with sympathetic, parasympathetic, and afferent nerves, leverages the intricate neural network of the target region to modulate cardiac function (Janes et al., 1986; Murphy and Armour, 1992; Kawashima, 2005; Wink et al., 2020; Zandstra et al., 2021). In addition, the target region for CPNS contains GPs (Chiou et al., 1997; Niu et al., 2007; Wang et al., 2010), which are home to a diverse array of neurons, including those forming local circuits (Armour, 1991; Armour et al., 1997). This variety of neurons within the GPs contributes to the nuanced and multifaceted effects of CPNS therapy. The effectiveness of CPNS is influenced by several factors, including the precise location of the electrode, stimulation parameters, patients's RPA neural anatomy, and the stage of CANS remodeling in HF patients. The positioning of the electrode in relation to specific nerves, especially those near the RPA like cardiac vagal nerves, plays a crucial role. For instance, if CPNS electrodes near cardiac vagal nerves are used, the therapy could induce bradycardia (Murphy and Armour, 1992). A multitude of afferent fibers are also present near the RPA which branch out toward either the dorsal root ganglia (DRG) or the nodose ganglia. This proximity of afferent fibers to the RPA underscores their potential involvement in the responses elicited by CPNS therapy (Janes et al., 1986; Murphy and Armour, 1992; Chiou et al., 1997; Kawashima, 2005; Niu et al., 2007; Wang et al., 2010; Wink et al., 2020; Zandstra et al., 2021). In this region, the presence of the RPA-GP or the superior vena cava-aorta (SVC-Ao) GP is notable. Stimulation of these GPs typically results in a deceleration of the sinus rate and atrioventricular conduction, demonstrating the profound impact of GP stimulation on cardiac rhythm (Cooper et al., 1980; Mick et al., 1992; Chiou et al., 1997; Lu et al., 2010). Simultaneously stimulating sympathetic, parasympathetic, and afferent fibers in CPNS can lead to non-specific CANS modulation, potentially causing issues like pain from direct nociceptive afferent stimulation or autonomic conflicts. This highlights the complexity and potential risks of CPNS, underscoring the need for precise modulation to avoid such adverse effects (Schwartz and Foreman, 1991; Chandler et al., 1993; Foreman et al., 2015; Eickholt et al., 2018; Winter et al., 2018).

The use of a catheter with multiple electrodes and the assessment of the hemodynamic response of stimulation seems to be essential in CPNS therapy for the accurate targeting of specific nerves. By selecting the electrodes adjacent to the targeted nerves, CPNS can precisely modulate nerve activity, thereby enhancing the therapy's effect and reducing the risk of non-specific modulation and related complications.

### Potential mechanism of action

It has been shown that low-level CPNS could provide significant benefits to ADHF patients (Emani et al., 2023). In this commentary article, we try to discuss the potential mechanism of action of CPNS, however, to determine the exact mechanism of action of CPNS, mechanistic animal studies are needed. In the recent CPNS clinical trial, the low-intensity CPNS was performed at a cardiopulmonary sympathetic nerve which was identified prior to initiation of CPNS therapy. By this method, the CANS is stimulated at a low level which does not cause a clinically relevant acute hemodynamic response. Although the low-level CANS modulation might not cause a large acute hemodynamic response, it can cause a profound protective effect by indirect modulation of the afferent pathway (Armour and Ardell, 2004). We hypothesize that during CPNS therapy, afferent signaling is modulated either directly by direct stimulation of afferent nerve fibers or indirectly by reacting to the low-level CPNS sympathetic stimulation. The CANS controls the sympathetic and parasympathetic outflow based on the afferent/sensory input and therefore afferent modulation could cause a significant change in the whole CANS (Chen et al., 2015; van Weperen and Vaseghi, 2023).

### Chronic effect

Neuromodulation therapies can provide therapeutic effects that last longer than the stimulation period as seen in the vagus nerve neuromodulation therapy in the setting of atrial fibrillation in which a 3 min vagus nerve stimulation provided more than 20 min of cardioprotective effect (Salavatian et al., 2016). The main reason for this prolonged therapeutic effect is that neuromodulation therapies aim to target the CANS imbalance, which is one of the root causes of HF progression.

Neuromodulation therapies, such as CPNS, have the potential to help the CANS exit the maladaptive state present during HF progression. This can lead to a more functional state that does not accelerate HF worsening and may even partially reverse it. The CANS is capable of adapting/changing its neural network to improve its processing function which ultimately results in an improvement of cardiac function. If the CANS, with the help of acute or chronic neuromodulation therapy, starts its own adaptive reverse remodeling process to reach a more functional state, cardiac function, and patient outcome can improve dramatically even without continuous neuromodulation therapy.

## Discussion

The malfunctioning CANS is heavily remodeled in cardiac diseases such as myocardial ischemia, myocardial infarction, heart failure, and arrhythmias (Francis and Cohn, 1986; Tjeerdsma et al., 2001; Campelo and Abreu-Lima, 2004; Piepoli and Capucci, 2007; Hoyer et al., 2008; Kardesoglu et al., 2011; Phillips, 2012; van Bilsen et al., 2017; Ali et al., 2018; Hadaya and Ardell, 2020; Elia and Fossati, 2023; Kumar et al., 2023). The CPNS is capable of modulating the entire state of the CANS through both direct and indirect afferent pathway modulation that is transmitted to multiple levels of the CANS. Therefore, such profound modulation of the CANS has the potential to cause a significant improvement in a patient's trajectory by restoring the autonomic balance as seen in the low level CPNS study (Abboud, 2010; Sobotka et al., 2013; Emani et al., 2023). Neuromodulation techniques can provide cardioprotective effects including, but not limited to, reduction in cardiac arrhythmia events, improvement of cardiac function, relief of symptoms like chest pain, shortness of breath, and palpitations due to cardiac disorders, and prevention of sudden cardiac death (Salavatian and Ardell, 2018; Hadaya and Ardell, 2020). By addressing one of the root causes of HF progression, this profound CANS modulation can promote long-term cardiovascular health and reduce the risk of recurrent cardiac events, rehospitalization, and mortality.

It is also important to note that the extent of the therapeutic effect of the neuromodulation therapies varies among patients and it depends on factors like underlying cardiovascular condition, CANS condition, the neuromodulation technique and its mechanism of action, and the overall health of the patient.

## Author contributions

SS: Conceptualization, Investigation, Supervision, Visualization, Writing—original draft, Writing—review & editing. JCS: Conceptualization, Funding acquisition, Investigation, Writing—original draft, Writing—review & editing. JAS: Conceptualization, Visualization, Writing—original draft, Writing—review & editing. IL: Conceptualization, Writing—original draft, Writing—review & editing. AM: Conceptualization, Writing—original draft, Writing—review & editing. JA: Conceptualization, Investigation, Writing—original draft, Writing—review & editing.
